# Improvement of small extracellular vesicle isolation from mouse model blood

**DOI:** 10.20517/evcna.2025.90

**Published:** 2025-12-24

**Authors:** Gloria Venturini, Antonella Ferrante, Nazzareno Di Carlo, Lucia Bertuccini, Francesca Iosi, Maria Condello, Alberto Martire, Federica Fratini, Zaira Boussadia

**Affiliations:** ^1^National Centre for drug research and evaluation, Italian National Institute of Health, Rome 00161, Italy.; ^2^Department of Life and Environmental Sciences, Polytechnic University of Marche, Ancona 60131, Italy.; ^3^Core Facilities, Microscopy Area, Italian National Institute of Health, Rome 00161, Italy.; ^4^Department of Neurosciences, Italian National Institute of Health, Rome 00161, Italy.

**Keywords:** Extracellular vesicles, mouse model, blood, size-exclusion chromatography, iodixanol gradient, proteomics

## Abstract

**Aim:** Small extracellular vesicles (sEVs) are membrane-bound nanoparticles secreted by virtually all cell types that have emerged as promising sources of protein biomarkers for a wide range of diseases, including central nervous system disorders. Blood sampling is the most informative and non-invasive biomarker source. Notably, mouse models represent essential systems for studying *in vivo* disease mechanisms and testing therapeutic strategies. Therefore, in this study, we investigated the suitability of two different isolation methods for sEV recovery starting from non-terminal mouse blood sampling, with the aim of identifying the most effective protocol for downstream biomarker discovery.

**Methods:** We performed and compared size exclusion chromatography (SEC) and ultracentrifugation followed by iodixanol density gradient (UC-IDG). Additionally, we optimized extracellular vesicle (EV) isolation from small-volume samples of both serum and plasma, since these represent the most used sources for *in vivo* preclinical biomarker research. Both methods were evaluated in terms of yield, purity, and EV protein content by nanoparticle tracking analysis, electron microscopy, and high-performance liquid chromatography-tandem mass spectrometry (HPLC-MS/MS) proteomics.

**Results:** SEC showed a higher number of isolated vesicles and EV-associated markers, while reporting a reduced percentage of blood-abundant co-isolated proteins, compared to UC-IDG. The use of plasma as a starting material resulted in a cleaner background, showing fewer protein aggregates. The obtained results emphasize the advantage of SEC in enhancing vesicle yield and purity levels.

**Conclusion:** This work contributes to sEV-derived biomarker research in mouse models by confirming plasma, rather than serum, as the most reliable source of EVs and providing evidence that SEC is more suitable than UC-IDG for EV isolation.

## INTRODUCTION

One of the greatest challenges for biomarker research is to exploit blood as a source, since it is a biological fluid easily accessible through a minimally invasive liquid biopsy. However, blood is a complex fluid-tissue, with approximately 50% of the volume composed of cells, a network of high-abundant proteins and lipoproteins, and many kinds of vesicles arising from all the body^[1]^. Extracellular vesicles (EVs) are a highly heterogeneous group of cell-derived membranous structures that are normally released by all cell types in the extracellular space. They are key mediators of intercellular communication and, following their release, they can fuse with the plasma membrane or be endocytosed into “recipient” cells, both at short and long distances^[2]^. It has been widely reported that EVs from endosomal origin take part in various biological processes across many species, and their secretion and content dynamically change during disease establishment and progression, providing crucial molecular information regarding the health status^[3]^. EVs carry and protect from degradation specifically loaded proteins, lipids, and nucleic acids, such as microRNA (miRNA), long non-coding RNA (lncRNA), genomic and mitochondrial DNA^[4,5]^. EVs were originally differentiated in exosomes, originating from endosomal processes within multivesicular bodies (MVBs) and released by fusion with the plasma membrane^[6]^, and ectosomes, which originate by direct budding of the plasma membrane into the extracellular space^[7,8]^. Nowadays, recent advances in EV research have revealed greater complexity and heterogeneity. In fact, novel populations of EVs and extracellular nanoparticles (ENPs) have been described, such as “mitovesicles” originating from mitochondria, “migrasomes” formed during cell migration, “exophers” involved in cellular homeostasis, “exomeres”, and “supermeres”^[9]^, and the list of EV subtypes continues to grow as the field progresses^[10]^. Due to the difficulties in assigning isolated EVs to a particular biogenesis pathway by the identification of specific biomarkers of subcellular origin, the most accepted nomenclature is based on dimensions, dividing vesicles between small (< 200 nm) and medium/large (> 200 nm)^[11]^. Small EVs (sEVs) remain the most studied, particularly the ones referred to as exosomes, whose endocytic biogenesis, contents, and functions are strictly regulated^[12]^.

EVs are ubiquitously present in several biological fluids, including saliva, plasma and serum, bronchioalveolar lavage, cerebrospinal fluid, amniotic fluid, breast milk, and urine^[13,14]^. Therefore, since biofluids can be collected via non-invasive or minimally invasive procedures, EVs have been proposed as a suitable source for diagnostic biomarker discovery^[15-17]^ as well as monitoring of treatment response in many pathologies and real-time evaluation of disease development^[18-22]^. The potential of EV-based liquid biopsy is still being explored, and the number of publications on EVs has increased in multiple scientific fields.

Plasma^[23]^ and serum^[24]^ represent the most commonly used starting materials to study blood EVs. Nevertheless, they can be considered as complex as a fluid tissue showing a high abundance of many different types of extracellular particles, leaking from all body districts, but also low-density cellular debris and highly-abundant protein and lipoprotein aggregates, which make it difficult to isolate pure EV populations^[25,26]^. Recent observations also highlight the presence of a protein corona that covers the surface of blood-derived EVs^[27]^, which is supposed to aid EV adhesion and uptake. Besides, the contamination of highly abundant blood proteins may mask specific EV marker identification^[28]^.

In the present study, we aim to establish the most suitable method for the isolation of sEVs to improve biomarker research in blood liquid biopsy from mouse models. In fact, although the interest in EV research has increased in the last 20 years, there are still no real gold standards in place to isolate different types of EV from blood. Therefore, we compared the two main substrates (plasma and serum) and two of the most used techniques for blood-derived sEV isolation^[29]^, i.e., size exclusion chromatography (SEC) and ultracentrifugation followed by iodixanol density gradient (UC-IDG).

To this aim, we used the blood collected from naïve C57Bl/6J mice. Even considering the limitations in recapitulating the human molecular, cellular, and clinical phenotypes^[30]^, mouse models still represent an important tool to elucidate the pathogenesis of diseases, and detect potential therapeutic agents^[31]^.

## METHODS

### Animals

All animal procedures were carried out in accordance with the European Community Guidelines for Animal Care (DL 26/2014), the European Communities Council Directive 2010/63/EU, and the FELASA (Federation of European Laboratory Animal Science Associations) and ARRIVE (Animal Research: Reporting of *in vivo* Experiments) guidelines. All procedures were approved by the Italian Ministry of Health (authorization no. 404/2023-PR) and by the Institutional Animal Care and Use Committee (IACUC) of the Istituto Superiore di Sanità (Rome, Italy).

Retro-orbital plexus blood sampling was performed on male and female C57Bl/6J mice [The Jackson Laboratory (JAX) strain #000664], aged 10-19 weeks, every 3 weeks, for a total of four samplings per animal. All collections were conducted in the morning between 9:00 and 11:00 AM.

The animals were kept in standard cages (48 cm × 26 cm × 20 cm, 4 mice per cage) under standardized temperature (22 °C), humidity (55%), with free access to water and food and lighting conditions (12:12 h light:dark cycle, with lights on at 6 AM and euthanasia/withdrawn of organs and blood sampling conducted as soon as possible after the start of the light phase). For animals used in the present study, proper treatment, care, and humane conditions have been provided. All efforts were made to reduce the number of animals used and to minimize their pain and discomfort. Permanent veterinary surveillance and animal welfare evaluation have been provided by the Host Institutions.

### Plasma separation

For plasma sampling, tubes were pre-treated with sodium citrate 3.8% (10×), and, after blood collection, tubes were gently rotated 8 times. Blood was processed within 30 min from sampling and following centrifuges, without brake, were performed: 600 × *g* for 15 min, 2,500 × *g* for 15 min, 5,000 × *g* for 15 min at 4 °C to remove cells, platelets, and debris respectively^[32]^. Supernatants in the last centrifuge were collected, leaving 1 cm over the pellet in the tube.

### Serum separation

Following sampling, blood was left for 1 h at room temperature (RT) for coagulation. Serum was then separated by centrifuging at 600 × *g* for 10 min at 4 °C. Supernatant was collected and centrifuged at 2,000 × *g* for 10 min at 4 °C in order to eliminate cell debris.

### Vesicles isolation

Plasma or serum derived from 7-8 animals was pooled together, and, before storage, two sequential centrifugations at 10,000 × *g* and 20,000 × *g* were performed to remove larger microparticles. Then, 5% dimethyl sulfoxide (DMSO) was added to support vesicle cryopreservation maintaining membrane integrity^[33,34]^, and samples were frozen at -80 °C in 1 mL aliquots until sEV isolation. Each biological replicate originated from a distinct group of animals. In total, three independent groups of 7-8 mice, each subjected to four blood samplings, were used for each biofluid. To minimize the impact of biological variability on the comparison between the two isolation methods, each plasma/serum pool obtained from a given group was divided into two equal aliquots, which were processed in parallel using the two vesicle isolation procedures. Animals were randomly assigned to the experimental groups, ensuring a balanced distribution of males and females across groups. Where “n” is reported in the figure legends, it refers to independent animal-derived sample pools.

#### Ultracentrifugation-iodixanol density gradient (UC-IDG)

For the isolation of sEVs, 1 mL of mouse plasma or serum was diluted with 0.22 µm filtered-phosphate buffered saline (PBS) to 5 mL and ultracentrifuged at 100,000 × *g* for 2 h, washed in PBS, and ultracentrifuged again at 100,000 × *g* for 2 h to remove any contaminants. Freshly pelleted sEVs were resuspended in 100 μL PBS, mixed with 1.4 mL of 60% (w/v) iodixanol (OptiPrep; Sigma-Aldrich, Saint Louis, MS, USA), and overlaid with 0.5 mL 40%, 1.5 mL 30%, 0.5 mL 10% and 1.5 mL 5% iodixanol. This protocol was modified starting from Kormelink *et al*.^[35]^, in order to isolate fractions with densities more aligned with those of EVs, while minimizing the inclusion of protein contaminants that tend to accumulate around 1.06 g/mL and exclude organelles and apoptotic bodies at higher densities. Afterward, sEVs floated into the gradient by ultracentrifugation (UC) in a SW55Ti rotor (Beckman) at 192,000 × *g* for 19 h at 4 °C. Gradient fractions of 500 μL were collected by pipetting from the top of the tube. The refractive index of each fraction was assessed with a refractometer (Carl Zeiss, Oberkochen, Germany), and the relative density was calculated using the linear relationship between refractive index (η) and the density (ρ) ρ = Aη - B. To remove iodixanol, fractions were then diluted to 1.5 mL, centrifuged for 40 min at 100,000 × *g*, and the pellets were resuspended in fresh, sterile-filtered PBS.

#### Size exclusion chromatography (SEC)

A qEV-35 nm column (Izon Science) was chosen as a commercially available column suitable for SEC and vesicle enrichment from plasma samples. Columns for 1 or 2 mL of starting material were tested with 1 mL blood ± 1 mL freshly filtered PBS dilution. In our hands, the 2 mL columns resulted in more reproducible vesicle isolation, maybe due to the 1:2 dilution before loading or to the higher number of chromatographic plates. The 2 mL column was used according to the manufacturer’s protocols. Briefly, after washing the column with PBS, samples were loaded and, after discarding the default buffer volume, 2 mL fractions were collected.

### Nanoparticle tracking analysis (NTA)

UC-IDG and SEC fractions were diluted as needed to obtain appropriate sample volumes and 20-120 particles/frame. Nanoparticle tracking analysis (NTA) was performed using an NS300 instrument (Malvern, UK; Software Version 3.4). The camera level was set to 14-15 for all recordings, and the camera focus was adjusted to make particles appear as sharp individual dots. Five 1-min videos were recorded for each sample at a syringe flow of 30 arbitrary units (AU). A detection threshold of 5 was used for analysis, with all other settings kept as default. After capture, the videos were analyzed using the built-in NanoSight Software NTA 3.4.4. In addition to mean, mode, particle concentration, and the typical D10 (10th Percentile), D50 (Median), and D90 (90th Percentile) values, we calculated the SPAN value. This represents a measure of the particle size distribution’s width, using (D90-D10)/D50, where D10 is the size where 10% of particles are smaller, D50 is the median (50% smaller), and D90 is where 90% are smaller.

After NTA, samples were recovered, centrifuged again at 100,000 × *g* in TLA100.1, and the pellet was used for proteomic analysis.

### Scanning electron microscopy (SEM)

sEV pellets suspended in PBS were left to adhere to polylisine-treated round glass coverslips for 4 h (50 µL). As previously described^[36]^, samples were fixed with glutaraldehyde 2.5% in 0.1 M Na-cacodylate buffer overnight (o.n.) at 4 °C and processed for SEM. Briefly, samples were post-fixed with 1% osmium tetroxide (OsO4) and were dehydrated through a graded series of ethanol solutions (from 30% to 100%). Then, absolute ethanol was gradually replaced by hexamethyldisilazane (HMDS) and, finally, samples were air dried at RT for 2 h, mounted on stubs, chrome sputtered (20 nm) by a TurboQ ES (Quorum Technology) and analysed in a field emission scanning electron microscopy (FE-SEM) Quanta Inspect F (ThermoFisher-FEI).

### Transmission electron microscopy (TEM)

sEV pellets suspended in PBS were analyzed by negative contrast staining. Samples were deposited on a copper grid with formvar/carbon support film. After standing for 15 min, the samples were dried with filter paper. Negative staining was carried out with 2% phosphotungstic acid (PTA) (pH 7.0) for 30 s. After blotting samples with filter paper, they were examined using an EM 208 FEI transmission electron microscope operated at 100 kV (FEI Thermo Fisher Scientific).

### Protein quantification

The protein quantification was performed using the Micro BCA^TM^ Protein Assay (Thermo Fisher Scientific, Rapid Gold BCA Kit; BCA, Bicinchoninic Acid) following default instructions. An undiluted 5 µL sample, lysed in 3% Sodium Deoxycholate (SDC) in 20 mM triethylammonium bicarbonate (TEAB), was used for quantification on the Qubit^TM^ 4 Fluorometer, and manufacturer’s instructions were followed.

### Silver Staining

First, 10 µL of each fraction were directly lysed and resuspended in an appropriate volume of sample buffer [100 mM tris(hydroxymethyl)aminomethane hydrochloride (Tris-HCl) (pH 6.8), 1% sodium dodecyl sulfate (SDS) 10% glycerol, 1% dithiothreitol (DTT) and blue bromophenol]. Then, 50 μM DTT was added, and samples were boiled for 5 min at 100 °C. Subsequently, sample proteins were resolved on 4%-20% SDS-polyacrylamide gel electrophoresis (SDS-PAGE), and silver staining kit (Pierce) was used to detect protein profiles, following user manual instructions.

### Proteomic analysis

Protein mixtures were reduced [10 mM tris(2-carboxyethyl)phosphine, 30 min at 56 °C] and alkylated (chloroacetamide, 40 min at RT in the dark) in solution; then diluted to 450 µL in 8 M urea in 20 mM TEAB and transferred to a Microcon 30-kDa centrifugal filter unit (Merck) previously washed with 0.5% SDC in 8 M urea, 20 mM TEAB. After buffer exchange, trypsin digestion (Sequencing grade modified trypsin, Promega) was performed in 0.5% SDC in 20 mM TEAB o.n. at 37 °C. Then, 50 μL of 20 mM TEAB were added to the filter unit and tryptic peptides were collected by centrifuging 5 min at 14,000 × *g*. The elution was repeated twice. Trifluoroacetic acid was added to a final concentration of 0.5% to stop the digestion and SDC precipitation. A final extraction with Ethyl-acetate 1:1 was performed twice to eliminate any salt trace, and then the peptide solutions were lyophilized in a SpeedVac, resuspended in buffer A [5% acetonitrile (ACN), 0.1% formic acid (FA)], and quantified by Quantitative Colorimetric Peptide Assay (Pierce). Each biological replicate was analyzed in technical triplicate (1 µg) using an Orbitrap Fusion Tribrid Mass Spectrometer (Thermo Fisher Scientific) coupled to an UltiMate 3000 Ultra High Performance Liquid Chromatography (UHPLC) system (Thermo Fisher Scientific). Peptides were firstly trapped in a μ-precolumn (C18 PepMap100, 5 μm, 100 Å, 5 mm × 300 μm, Thermo Fisher Scientific) and then run on a home-packed 25 cm × 75 μm id fused-silica column (8 PicoTip Emitter, New Objective, Littleton, MA, USA) packed with ReproSil-Pul C18-AQ 1.9 µm beads (Dr. Maisch GmbH, Ammerbuch, Germany) for chromatographic separation. Peptides were eluted at 0.2 μL/min along a 120 min linear gradient from 8% to 35% buffer B (95% ACN, 0.1% FA). Full-scan mass spectrometry (MS) was acquired in Orbitrap at 60 K resolution - maximum injection time of 50 ms, 1 microscans, wide quadrupole isolation activated in a mass range of 350-1,550, and an AGC target of 4E5. The MS/MS scans were automatically acquired in the ion trap for a total cycle time of 3 s; quadrupole isolation window 1.6; minimum intensity 5E3; HCD fragmentation; NGC 32; normal scan rate; maximum injection time of 35 ms; AGC 5E3. Dynamic exclusion allowed a repeat count of 1 within 45 s; max tolerance 10 ppm. Spectra raw files have been processed by MaxQuant 2.4.2, using Mouse_ReferenceProteome_2023_10090 and Uniprot_Musmusculus_Database_2022 for search engine, including the following setting: carbamidomethylation as fixed modification; methionine oxidation; N-term acetylation and Serine, Threonin, Thyrosin (S,T,Y) phosphorylation as variable modifications; maximum 2 missed cleavage were allowed; False Discovery Rate (FDR) was set to 0.01% for Peptide Spectrum Match (PSM) and protein identification and a minimum of 2 ratio count was set for Label Free Quantitative (LFQ)^[37]^. The identified and quantified protein dataset has been investigated by Perseus 2.0.10.0. Proteins identified as reverse or only identified by site were filtered out, and only proteins quantified in at least 2/3 biological replicates were considered for further analyses. Two-sample Student’s *T*-test (SEC *vs*. UC-IDG) was performed by using the recommended settings of S0 = 1.5 and FRD = 0.01, where S0 is a variance regularization parameter, which controls the relative importance of t-test p-value and difference between means among biological replicates^[38]^. Raw files, Databases, MaxQuant search engine result files, and Perseus plain matrix result files are available at Massive (MSV000098475) and ProteomeXcharge (PXD066022) repositories.

### Western blotting

Isolated EVs were pelleted, and radioimmunoprecipitation assay (RIPA) Buffer with freshly added protease and phosphatase inhibitors (Thermo Fisher Scientific) was added, and samples were kept on ice for 30 min. Protein extracts were resuspended in Tris-Glycine SDS Sample Buffer containing Sample Reducing Agent at a final 2× concentration, and heated for 10 min at 100 °C. Samples were then separated on precast Novex Tris-Glycine Mini Gels. Proteins were transferred using iBlot^TM^ 2 on a polyvinylidene difluoride (PVDF) membrane, which was then blocked in 5% nonfat dry milk (Biorad Laboratories) in Tris-buffered saline with Tween® 20 detergent (T-TBS) for 1 h at RT. Membranes were then incubated o.n. with anti-tumor susceptibility gene 101 (Tsg101) (EPR7130) rabbit monoclonal antibody (1:1,000, cat#ab125011, Abcam), anti-heat shock protein 90 (HSP90) (C45G5) rabbit monoclonal antibody (1:1,000, cat#4877, Cell Signaling), and anti-cluster of differentiation 81 (CD81) (D5O2Q) rabbit monoclonal antibody (1:1,000, cat#10037, Cell Signaling) in T-TBS with 5% nonfat dry milk. After incubation, membranes were washed three times in T-TBS for 10 min at RT and incubated for 1 h with anti-rabbit horseradish peroxidase-conjugated antibodies (1:10,000, cat#111-035-003, Jackson Immunoresearch Laboratories) at RT. Reactivity was detected using WesternBright enhanced chemiluminescence (ECL) spray device (cat#K-12049-D50, Advansta, Aurogene) and Chemidoc (Biorad).

### Statistics

Statistical analysis was performed by using t-test, and normal distribution was verified using Shapiro-Wilk test. Statistical analyses and curve fittings were obtained by using GraphPad Prism software (version 9, GraphPad Software, San Diego, California, USA). All data are reported as mean ± standard error of the mean (SEM) of different independent experiments as indicated in the Results section or in Figure legends. Differences were considered statistically significant at *P* < 0.05 (^*^P  < 0.05; ^**^ *P* < 0.01; ^***^P < 0.001).

### EV-track

All detailed methods and data were submitted to the EV-TRACK knowledgebase (ID: EV250075)^[39]^.

## RESULTS

### Evaluation of quality and quantity isolation yields

**Figure 1:**
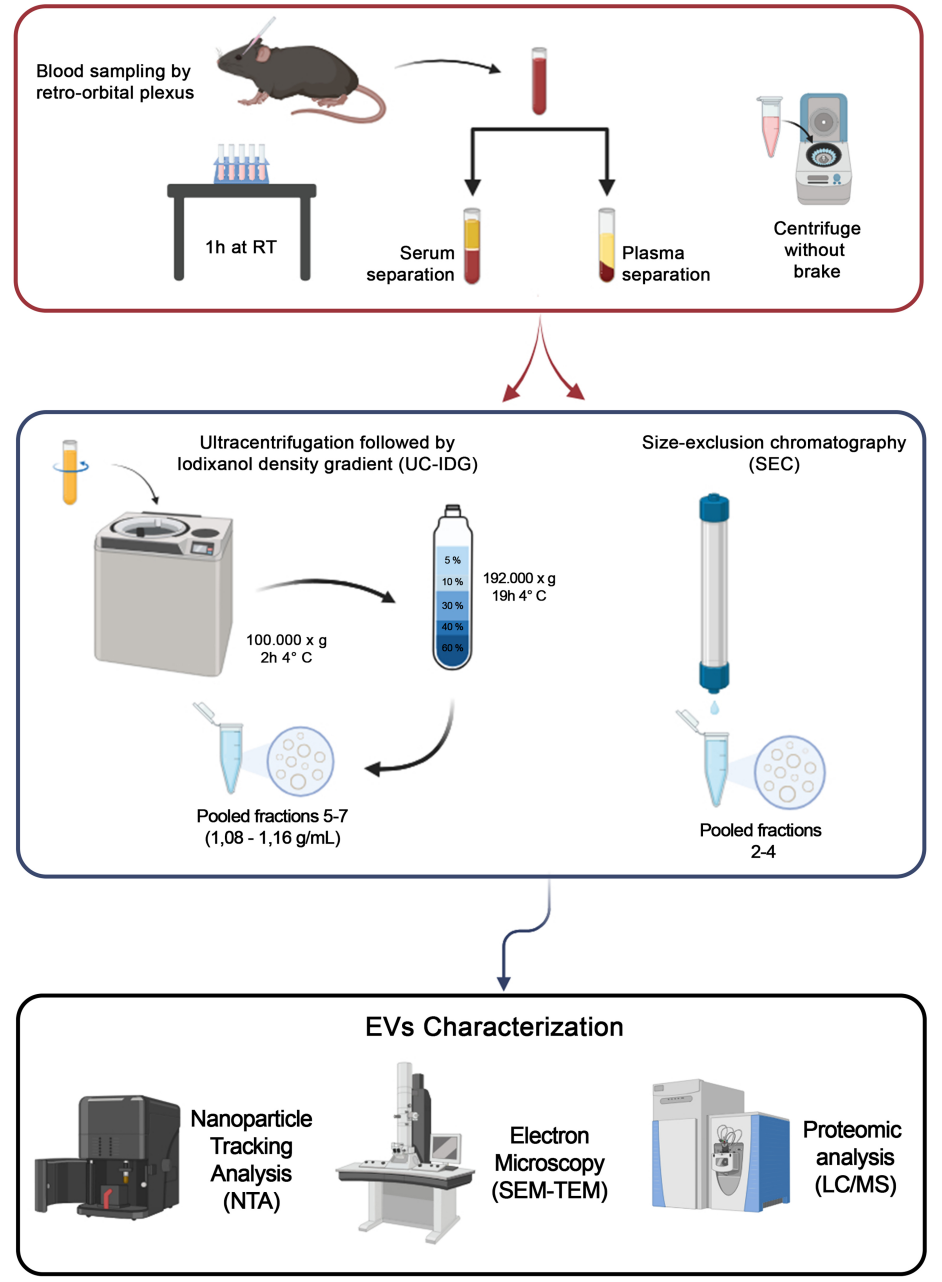
Workflow of the sEV isolation protocol. Blood was obtained by retro-orbital plexus puncture and separated into plasma or serum. For sEV isolation, two methods were used: Size exclusion chromatography (qEV 2 Izon 35 nm) and ultracentrifugation at 100,000 × *g* followed by a bottom loaded iodixanol density gradient (5%-60%). Resulting samples were counted by NTA, visualized by SEM and TEM, and protein content analyzed by Mass Spectrometry. Created in BioRender. Boussadia, Z. (2025) https://BioRender.com/n7dp03u. sEV: Small extracellular vesicles; NTA: nanoparticle tracking analysis; SEM: scanning electron microscopy; TEM: transmission electron microscopy; LC/MS: liquid chromatography/mass spectrometry; RT: room temperature; UC-IDG: ultracentrifugation followed by iodixanol density gradient.

The complete workflow is shown in Figure 1. Blood sampling (ca. 200 µL) from 7-8 mice was immediately processed for plasma/serum separation, and pooled to obtain the minimum volume of 1 mL of starting biofluid for each methodology. Then, vesicles were isolated either by UC-IDG or SEC. Size and concentration of each fraction collected by both methods were characterized by NTA. Then, fractions were pooled together, and the vesicle morphology was analysed by SEM and TEM. Finally, proteomics characterization was performed.

**Figure 2:**
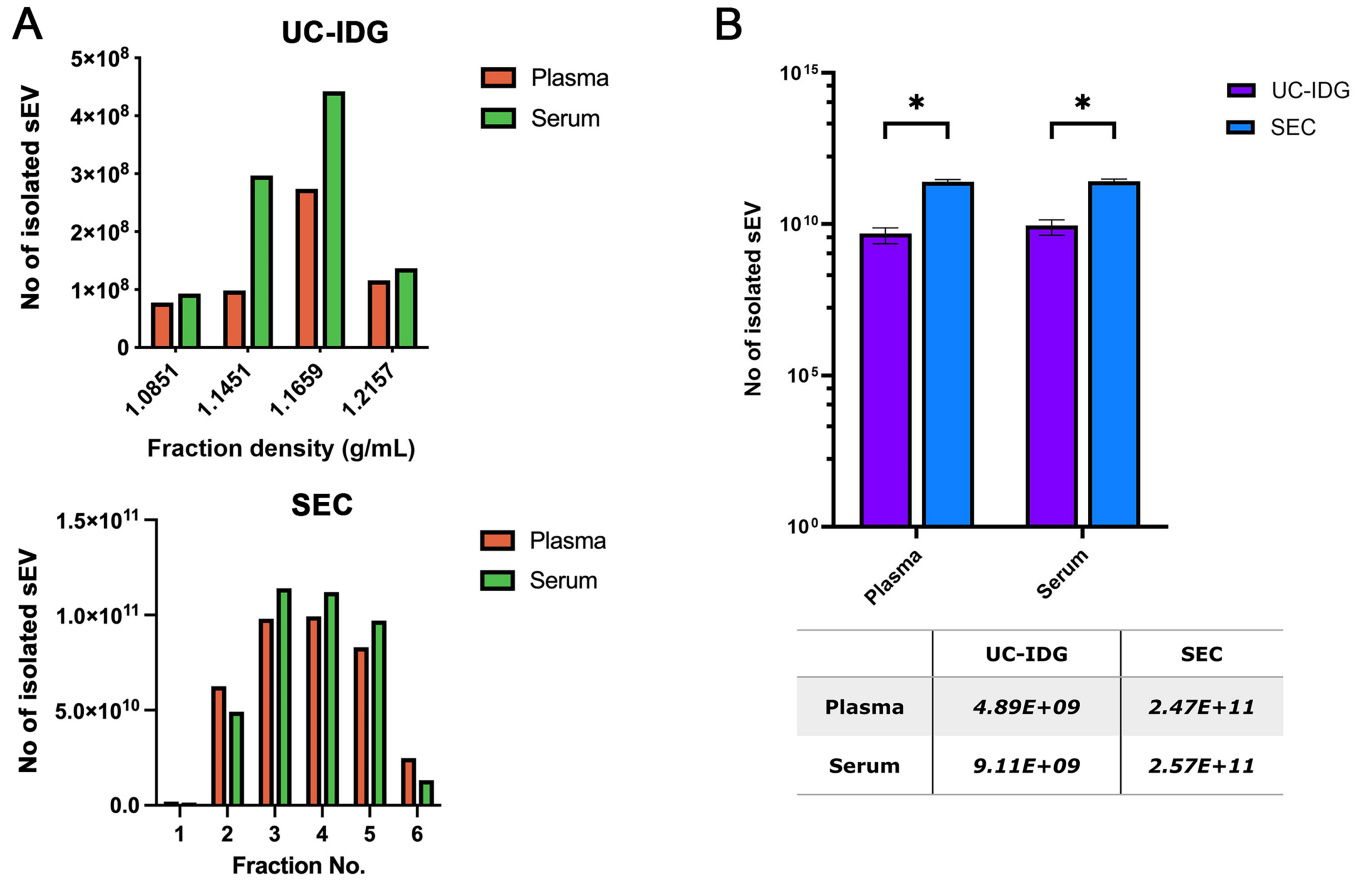
Isolation yields by nanoparticles tracking analysis. (A) Particle concentration analyzed by NTA of UC-IDG fractions (5-8) and SEC fractions. The number of counted particles in pooled fractions is represented and compared in (B). Bars are on a logarithmic scale. Single values are reported in the underlying table. Data presented as mean ± SEM; *n* (independent biological replicates) = 3. ^*^P < 0.05. UC-IDG: Ultracentrifugation followed by iodixanol density gradient; SEC: size exclusion chromatography; NTA: nanoparticle tracking analysis; SEM: standard error of mean.

As shown in Figure 2A, the two biofluids contain a comparable number of vesicles, and they were enriched in the same fractions both in UC-IDG and SEC preparation. The total number of isolated particles by SEC was 50-fold higher (ca. 2.5E10^11^) compared to UC-IDG (ca. 5-9E10^9^), for both plasma and serum [Figure 2B].

**Figure 3:**
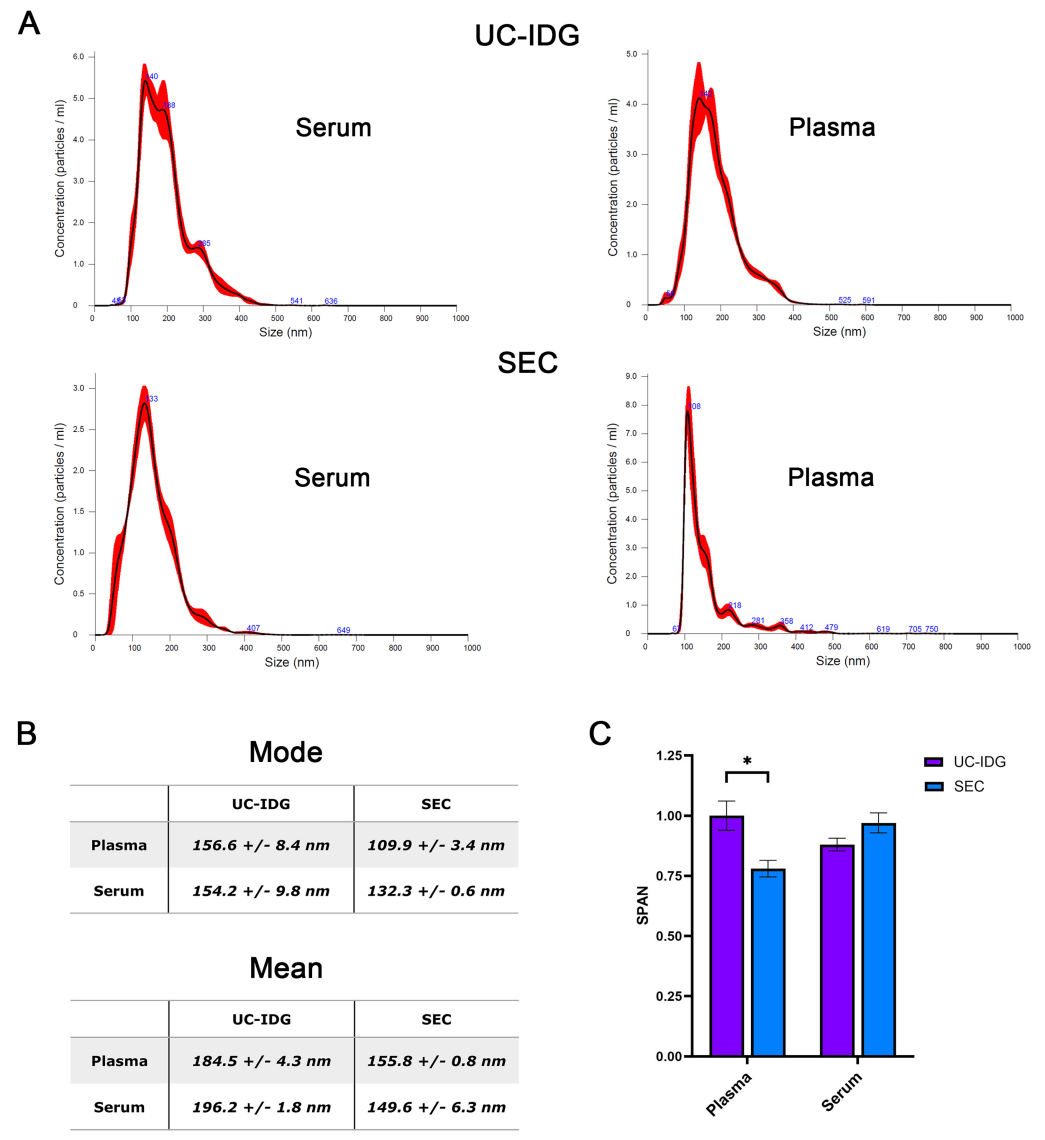
Particle distribution evaluated by NTA. (A) Size distribution in the most represented fraction (Fr. 1,1659 g/mL for UC-IDG and fraction 3 for SEC) as assessed by NTA. The mean and the median particle size are represented in (B). SPAN, derived from NTA size distribution values [(D90-D10)/D50] was calculated for each sample and represented in (C). Data presented as mean ± SEM; *n* (independent biological replicates) = 3. ^*^P < 0.05. UC-IDG: Ultracentrifugation followed by iodixanol density gradient; SEC: size exclusion chromatography; NTA: nanoparticle tracking analysis; SEM: standard error of mean.

Hereafter, we assessed particle size distribution by looking at NTA profile [Figure 3A and B]. As expected, SEC purifies a more homogeneous population, resulting in a thinner peak and a smaller mode size (132.3 ± 0.6 nm for serum and 109.9 ± 3.4 nm for plasma) compared to UC-IDG (154.2 ± 9.8 nm for serum and 156.6 ± 8.4 nm for plasma). Further, the SPAN value, measuring the size distribution, results significantly lower for SEC isolation from plasma, while no significant differences were reported for serum [Figure 3C].

**Figure 4:**
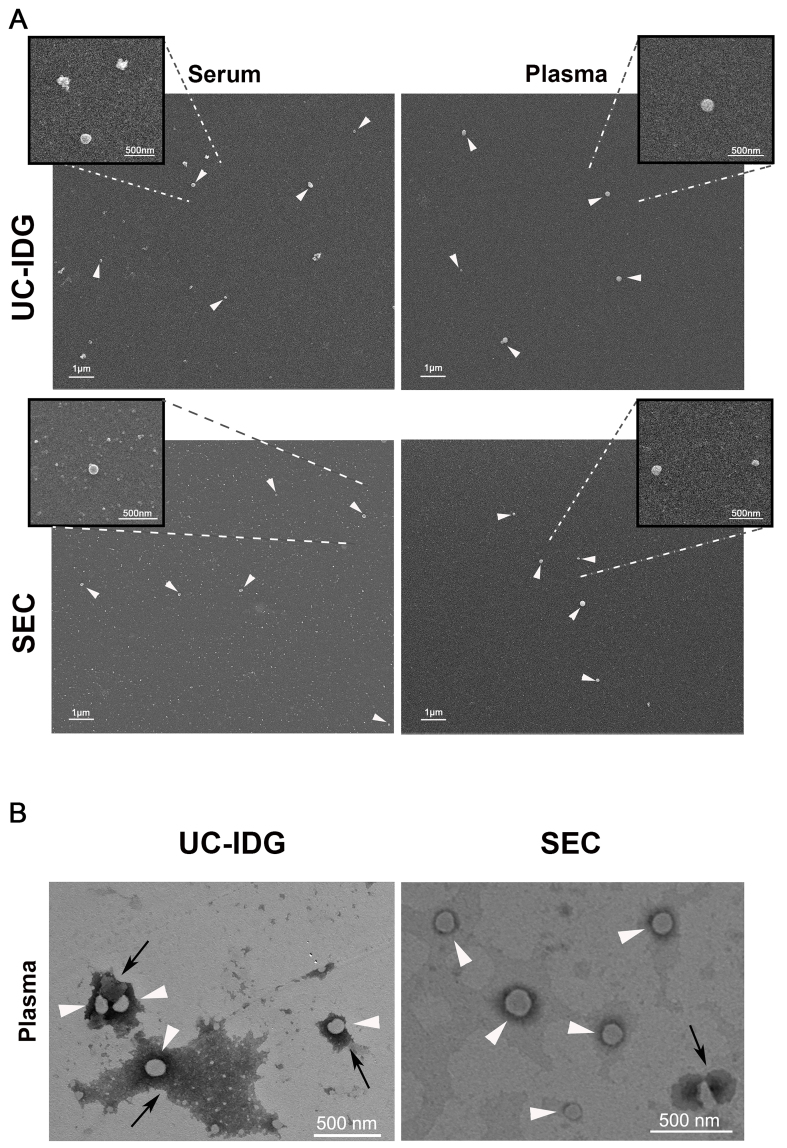
Evaluation of sEV morphology by electron microscopy. (A) SEM micrographs of sEV pellets derived from serum or plasma samples, isolated by UC-IDG or SEC (scale bar 1 µm). Inserts represent higher magnification images of the selected region of interest, showing sEVs (scale bar 500 nm); (B) TEM observation of vesicles obtained by plasma UC-IDG and SEC showing the lipid bilayer-enclosed structure (white arrows) and protein aggregates (black arrows). UC-IDG: Ultracentrifugation followed by iodixanol density gradient; SEC: size exclusion chromatography; sEV: small extracellular vesicle; SEM: scanning electron microscopy; TEM: transmission electron microscopy.

Further, to characterize particle surface morphology and purity, we performed electron microscopy experiments using a Field Emission Scanning Electron Microscope [Figure 4A]. Results indicated that both methods compared in this study led to the obtaining of EV populations characterized by the presence of small vesicles adhering to polylysine round glass coverslips, with both serum samples emerging from a higher background of protein aggregates compared to those from plasma [Figure 4A]. Although SEM analysis does not allow a quantitative assessment of the yield of the two methods, adhered vesicles are more easily found in the samples obtained by SEC.

To confirm the lipid bilayer-enclosed structures of the vesicles, we further analyzed the plasma sample by TEM [Figure 4B], which showed clear spherical vesicles of a size comparable to the NTA results and confirmed a higher amount of protein aggregates in UC-IDG samples compared to SEC.

Therefore, even if we observed no differences in particle concentration and size by NTA that would indicate one biofluid as better than the other, the sharper peak in NTA plasma samples - particularly in the SEC preparation - suggested a more homogeneous population. This was further confirmed by electron microscopy, which showed a higher background in serum samples compared to plasma. Thus, considering these results, the more controlled and reproducible procedure for obtaining plasma relative to serum, and also taking into account what is already known in the literature - that during the clotting period platelets are activated and secrete large amounts of vesicles, introducing a concentration bias in the comparison^[24]^ - we finally concluded in favor of plasma samples for subsequent investigation of protein cargo.

### Proteomic analysis of EV markers

For proteomics, we pooled the sEV-enriched fractions arising from UC-IDG and SEC, respectively. For UC-IDG samples, we pooled fractions 5-7 (1.08-1.16 g/mL), while for SEC samples, we selected fractions 2-4 because, although fraction 5 contains a large number of particles (as shown by NTA), Silver Staining experiments also revealed a high concentration of proteins [Supplementary Figure 1], attributable to blood contaminants that could interfere with the analysis of sEV protein content.

**Figure 5:**
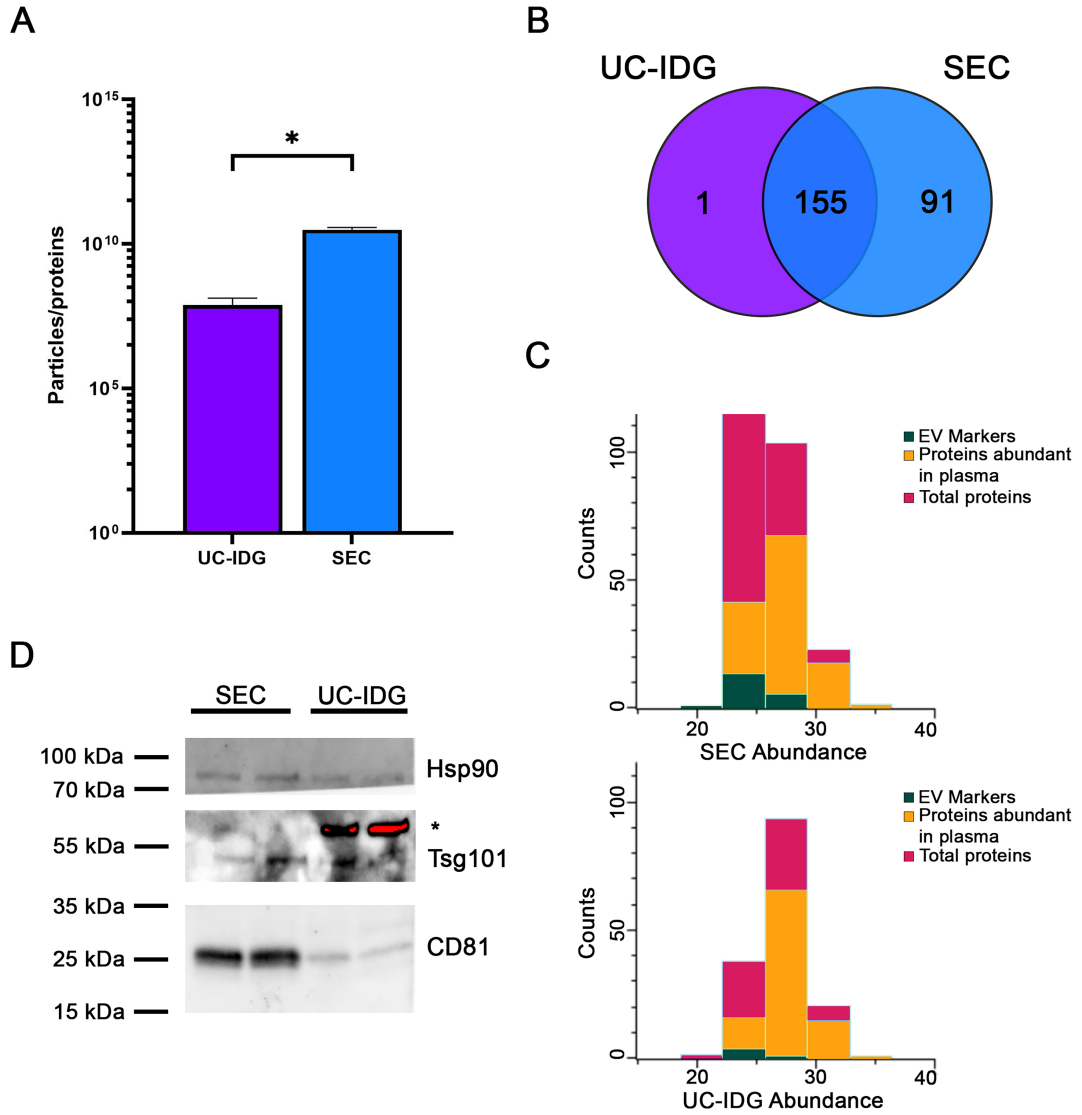
Proteomic analysis by Mass Spectrometry. (A) Graph shows particles per protein, obtained by dividing the number of particles counted by NTA by the protein amount measured using BCA. This ratio is an indication of sample purity; (B) Venn diagram analysis of proteins identified by Mass Spectrometry of sEVs isolated by UC-IDG and SEC; (C) Representation of proteins profiled by LC/MS. In red are total proteins; green the EV markers; in orange the plasma abundant proteins. For all graphs, data is presented as mean ± SEM; *n* (independent biological replicates) = 3. ^*^P < 0.05; (D) Representative image of western blot analysis of SEC and UC-IDG samples showing levels of some known and accepted EV markers. ^*^indicates an unspecific band. NTA: Nanoparticle tracking analysis; BCA: bicinchoninic acid; sEVs: small extracellular vesicles; UC-IDG: ultracentrifugation followed by iodixanol density gradient; SEC: size exclusion chromatography; LC/MS: liquid chromatography/mass spectrometry; EV: extracellular vesicle; SEM: standard error of mean.

Samples were lysed, protein contents were quantified, and the ratio to the number of particles counted by NTA was evaluated as an indication of sample purity^[40]^. Indeed, the major challenge for vesicle purification from blood samples is an efficient separation from high-abundant protein aggregates, mainly immunoglobulins, complement factors, albumin, *etc*. The particle/protein ratio, derived from NTA-based vesicle quantification and total protein concentration, provides a useful index of vesicle purity by normalizing vesicle counts to protein content; in fact, a higher ratio is an indirect index of fewer particles formed by protein aggregates. Particles/protein were higher in SEC, thus suggesting a lower amount of protein contaminants [Figure 5A], confirming a better resolution capability of SEC *vs.* iodixanol gradient.

Nevertheless, for a deep characterization of sample preparations, we performed a proteomic profiling by MS. In line with the higher concentration measured by NTA, proteomics analysis identified more proteins in SEC sample (246) *vs*. UC-IDG (156). Mostly UC-IDG proteins were common to SEC, which enriched 91 proteins more [Figure 5B]. All protein annotations and MS features are reported in Supplementary Table 1.

To highlight any quantitative difference in proteins identified by both methods, we performed the two-sample Student’s *T*-test (SEC *vs.* UC) using the Perseus platform [Supplementary Figure 2]. The statistical comparison shows no differences in quantitative abundance of proteins identified in the two samples, except for a few apoliproteins (A4, C3, E) in SEC sample - known to be part of the vesicle-corona - and blood contaminants (Trypsin and Desmoplakin) in UC-IDG sample. Thus, it confirmed that the differences in the two methods do not belong to vesicle content but to the vesicle/aggregates recovery amount. Indeed, to gain an overall understanding of the protein composition of the two samples, a manual annotation, based on accumulated authors’ experience and previous literature, was carried out to highlight main “proteins abundant in plasma” (such as hemoglobin, albumin, immunoglobulin, *etc*.) and sEV markers^[25,28,34,41]^ [Figure 5C]. SEC resulted in a higher yield of vesicle isolation, allowing the identification of a higher number of proteins and sEV markers, improving the ratio over blood contaminants (4.5% in UC-IDG *vs.* 11.4% in SEC). Also, 34 proteins (13%) in SEC *vs.* only 12 proteins (8%) in UC-IDG were annotated in the Top 100 EV Proteins listed in Exocarta database (http://www.exocarta.org/) as reported in Supplementary Table 1. We then performed western blot analysis to evaluate the presence of well-established sEV markers in both samples [Figure 5D]. As expected, the markers CD81, Hsp90, and Tsg101 were present in both samples but appeared more abundant in SEC compared to UC-IDG.

### Gene ontology characterization

**Figure 6:**
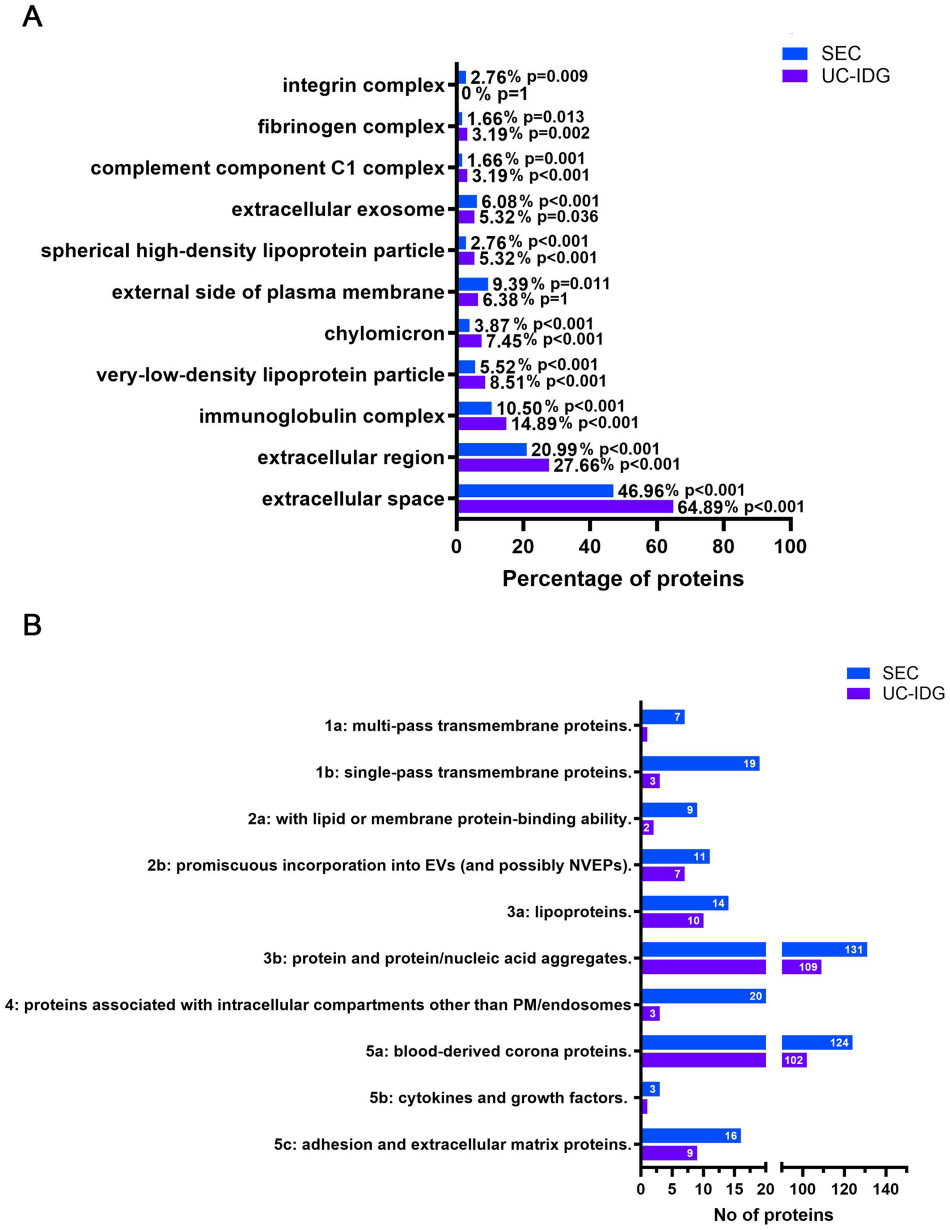
Enriched Gene Ontology terms among identified proteins. (A) Results of the analysis of cell compartments GO enriched in the two samples, following protein identification by LC/MS; (B) Proteins were categorized according to MISEV2023 guidelines, and the graph reports the number of proteins in each category for SEC and UC-IDG. GO: Gene ontology; LC/MS: liquid chromatography/mass spectrometry; MISEV: Minimal Information for Studies of Extracellular Vesicles; SEC: size exclusion chromatography; UC-IDG: ultracentrifugation followed by iodixanol density gradient.

To further characterize sEV content, Cellular Component Gene Ontology (GO) enrichment analysis was performed [Supplementary Table 2], and the most interesting and significant categories are listed in Figure 6A. Considering the total number of identified proteins for each purification method, SEC was enriched in categories such as “integrin complex”, “extracellular exosome”, and “external side of plasma membrane”, which are closely related to EVs. In contrast, UC-IDG was enriched in categories associated with blood contaminants, including fibrinogen, high density lipoprotein (HDL), very low density lipoprotein (VLDL), and chylomicrons [Figure 6A].

“Extracellular exosomes” was annotated for 6.8% and 5.32% in SEC and UC-IDG, respectively. As expected, “Extracellular region” and “extracellular space” were the most abundant categories [Figure 6B] and were more represented in UC-IDG. These GO terms are mostly overlapping and include secreted proteins, soluble factors and signaling molecules, proteins found in plasma or serum, and ECM components present in the intercellular space. As such, these categories include a mix of possible EV-specific proteins, EV-corona proteins, and probably blood-derived protein aggregates. The higher enrichment of these categories in UC-IDG, despite the lower number of identified proteins, further confirms the better quality of SEC isolation.

Additionally, to gain deeper insight into the composition of the two samples, we classified proteins based on Minimal Information for Studies of Extracellular Vesicles (MISEV2023) guidelines [Figure 6B]. Results reflected the higher abundance of identified proteins in SEC, compared to UC-IDG, highlighted by all previous analyses. All categories were found to be more enriched in the SEC fraction. The annotation into MISEV2023 of all the identified protein datasets [Supplementary Table 1] resulted in evidence that not only does SEC isolation yield a better quantity, but also a higher quality purification. In fact, considering categories 1, 2, and 5 as representing the most reported EV proteins, and categories 3, 4, and overlapping 3a/b, 5a (i.e., blood contaminants overlapping with corona proteins), we found that 30% of proteins identified in SEC bona fide belonged to sEVs, compared with only 18% in UC-IDG preparations.

We further validated our initial classification of sEV markers and plasma proteins, examining the MISEV2023 categories to which they belong [Supplementary Figure 3].

We found that proteins initially annotated as EV markers mainly fell into categories 1 and 2, with a small subset in category 5c. In contrast, proteins initially classified as abundant plasma components were primarily associated with categories 3 and 5a. These observations support the accuracy of our initial categorization.

## DISCUSSION

One of the main prerequisites for studying sEVs in cell-cell communication, and as a reservoir of potential diagnostic and prognostic clinical biomarkers, is a suitable sEV isolation method allowing the discrimination of sEVs from contaminants. When using blood as a starting material for the identification of reliable blood-based EV liquid biopsy biomarkers, the challenge is exacerbated by the presence of highly abundant blood contaminants and several kinds of particles^[42]^. Even if a relevant guideline from the International Society of Extracellular Vesicles has been published^[43]^, there is still no consensus regarding an optimal preparation method, with the only recommendation available for using the most appropriate method according to the planned downstream application and the biological source.

Even if human plasma has been widely studied and frequently used, the literature is full of proteomics analysis containing blood contaminants as vesicle cargo; furthermore, the use of plasma derived from mouse models is still poorly investigated even if it could be of great support in preclinical research. Even more, limited data are available comparing techniques for EV isolation and characterization starting from mouse biofluids.

A recent study aiming to identify biomarkers in non-obese diabetic mice compares the SEC technique with membrane affinity (exoEasy/MA). The study demonstrated that different EV isolation methods can lead to distinct proteomic profiles, with SEC preparations providing proteomes less overlapping with whole plasma depleted of abundant proteins and therefore enriching for a more specific sEV proteome^[44]^. There are several methods to isolate sEVs^[45,46]^; however, UC-based techniques have represented the “golden standard” for years. Additionally, the availability of a large amount of sample volume and access to UC is a major limitation in the preclinical research and setting of this protocol and, more importantly, as our study confirms, proteomics analyses revealed the scarce quality of sEV enrichment over plasma/serum contaminants^[47]^.

Therefore, in the present study, we compare UC-IDG and SEC for the isolation of sEVs from non-terminal mouse-derived blood and we carefully characterized quality and quantity yields in terms of size distribution, concentration and protein content. The introduction of a density gradient following the UC improves the isolation quality over UC alone but results in a longer protocol and loss of yield. Although SEC has emerged in the last years as an alternative to UC for sEV isolation, early comparative studies reported moderate yield and technical limitations^[48-51]^. The recent availability of a new generation of SEC columns, including those used in this study, prompted us to compare this isolation strategy with the UC-IDG protocol employed in our laboratory to identify the optimal procedure for downstream proteomic and transcriptomic analyses. From an overall perspective, SEC columns need low infrastructural requirements and are easy to use, making them suitable for applications in settings where other methodologies would be prohibitive, thus providing a promising approach for extending the scope of extracellular research beyond the laboratory, into both preclinical and clinical applications.

In our study, SEC led to the greatest yield as assessed by NTA and, analyzing SPAN, a measure of particle size distribution, SEC isolates a significantly more homogeneous vesicle population in plasma sample. This is probably due also to a higher probability of vesicle merging and vesicle/protein-aggregates recovery by UC, and a worse resolution capability of iodixanol density gradient *vs.* SEC. Electron microscopy experiments, while confirming the membrane-bound morphology of sEVs in all samples, also highlighted the presence of a higher background in serum samples compared to plasma, according to a wider size distribution in NTA. We hypothesize a higher variability of serum-derived sEV population, partly due to the uncontrolled aggregation during the coagulation step and partly to the known release of sEVs from platelets^[52,53]^. Therefore, our experimental evidence, although not showing a difference in yield between the two starting biofluids, suggests a higher purity of plasma samples, confirming previously reported literature that identifies plasma as a more suitable source for a standardized protocol. Thus, we finally selected plasma as a blood-derived starting material for sEV isolation and liquid-biopsy biomarker research.

As for the evaluation of widely discussed sEV sample purity, it was initially hypothesized that a pure population of 10^10^ sEV would theoretically contain ~1 μg of protein or even less^[40,54]^. Nevertheless, with samples derived from plasma or serum, such purities are difficult to achieve^[40]^. Calculating particle-to-protein ratio suggests that SEC can approach this theoretical approximation. This confirms that SEC purification of EVs reduces plasma protein contamination. However, it is increasingly clear that the particle-to-protein ratio provides only an estimate of plasma sEV purity, and that proteomic analysis is required to rigorously define protein content and sEV purity.

When analyzing MS data, the higher particle yield identified by NTA using SEC was confirmed by a greater number of identified proteins and EV-related markers. Multiple authors reported the co-purification of lipoproteins and sEVs using SEC, which appears to be an inherent property of SEC^[55]^, given the overlap in size between sEVs and very low-density lipoproteins and chylomicrons^[56]^. However, our results showed a lower enrichment of these categories in SEC compared to UC-IDG. Furthermore, we can argue that the presence of residual co-isolating proteins seems comparable between the two techniques; this is mostly ascribable to the corona-proteins, considering the higher concentration of isolated sEVs by SEC. Recently, the EV corona has become an increasingly central topic of debate^[27]^. It is made up of proteins and particles that directly interact with EV surface, playing a key role in mediating their transfer to target cells^[57,58]^. Here we found that proteins potentially belonging to the corona and following in the overlapping MISEV2023 categories 3a/b; 5a are slightly differentially enriched in SEC, as expected by the higher concentration of vesicles resulting from this protocol.

To get insight into the origin of proteins identified by high-performance liquid chromatography-tandem mass spectrometry (HPLC-MS/MS), GO cell compartment was investigated, and, also, identified proteins were classified by the functional categories described in MISEV2023. In line with previous results, SEC identifies a higher number of sEV-related proteins, compared to UC-IDG. In general, the increased ratio in SEC between EV-specific proteins and contaminants derived from blood or from organelles - not involved in sEV biogenesis, secretion, or degradation - suggests a reduced level of contamination and a more accurate isolation of EVs using SEC.

The high protein abundance and dynamic range of blood make this biofluid one of the most difficult to work with in MS-based proteomic studies. Overall, our findings indicate that when working with a limited volume of biofluids, SEC results in a higher quality and quantity of sEVs than UC-IDG and that this assay yields deeper proteomic coverage even with such a small starting material. Studies on human plasma have consistently demonstrated that isolation methods such as SEC and immune or non-immune affinity-based approaches provide higher purity of vesicular proteins compared to other techniques. These methods are therefore increasingly preferred for proteomic biomarker discovery, as they minimize protein contamination and co-isolation of non-vesicular material^[59]^. Nevertheless, achieving complete separation of EVs from plasma-derived contaminants remains a major challenge, largely due to the intrinsic complexity of the plasma matrix. The choice of isolation technique strongly influences downstream analyses, as the presumed high-yield methods, such as UC, are widely adopted for their simplicity and ability to recover large amounts of vesicles and high protein yield, but they are much more prone to co-isolate blood abundant contaminants^[26]^. Nevertheless, only by electron microscopy and above all proteomics investigation is it possible to certainly discriminate vesicle enrichment. Although combining multiple isolation approaches may enhance sample purity, this often comes at the expense of lower recovery, higher variability, and longer processing time. In contrast, SEC has resulted in a rapid and easy methodology suitable for getting a remarkable yield of fairly clean vesicles. In conclusion, the choice of the most suitable separation method must be based on the enrichment of sEVs and depletion of different contaminants that it allows, and it depends on the purity required for the desired application. In this study, we suggested SEC as a valuable methodology for plasma sEV enrichment and biomarker research for monitoring progression and therapeutic response in mouse models of diseases.

### Limitations of the study

This study has some limitations that should be noted. First, the critical challenge in EV research is the ability to firmly characterize the vesicle populations recovered by any preparation strategy. Even by proteomics analysis it is not possible to discriminate the presence of different vesicle populations, and any isolation strategy may selectively enrich specific subtypes. Consequently, not only particle number but also protein content and concentration may reflect the vesicle population isolated by each methodology, thus affecting the interpretation of comparative results. To mitigate this issue, we combined multiple analytical approaches to strengthen the evaluation of isolation performance. Another main limitation of the study refers to the limited amount of starting material, derived from a non-terminal mouse blood sampling. Actually, the aim of the study is to provide the most suitable strategy to overcome this limitation. The first step was to use a pool strategy to get enough volume in a suitable time. For a non-terminal blood sampling, a maximum of 200 µL of blood can be taken from each mouse every 3 weeks. Pooling samples allowed us to obtain the amount of blood and vesicles required for different preparation strategies and proteomics analyses, respectively, in a reasonable time. On the other hand, this could affect comparisons between biological replicates, which is why great care was taken to prepare each pool from different mice, so that each could be considered an artificial single-mouse biological replicate, accounting for the most representative proteomic profile of a healthy mouse population. Moreover, by pooling different mice, individual proteome variability is minimized, making proteomics statistical results stronger within the same number of biological replicates analyzed. Nevertheless, the yield of vesicles obtained by UC-IDG from 1 mL of plasma resulted in a limited proteome coverage in respect to SEC, thus presenting more than one third less identification which limited the statistical quantitative comparison. Increasing the initial plasma volume could improve data quality, especially when comparing multiple samples for biomarker discovery purposes.

## DECLARATIONS

### Availability of data and materials

Raw files, Databases, MaxQuant search engine result files and Perseus plain matrix result files are available at Massive (MSV000098475) and ProteomeXcharge (PXD066022) repositories. Methods regarding EV isolation have been submitted to EV-Track knowledgebase (ID: EV250075). All data that support the findings of this study are available from the corresponding author upon reasonable request.

### Copyright

© The Author(s) 2025.

## References

[1] Arraud N, Linares R, Tan S (2014). Extracellular vesicles from blood plasma: determination of their morphology, size, phenotype and concentration. J Thromb Haemost.

[2] Abels ER, Breakefield XO (2016). Introduction to extracellular vesicles: biogenesis, RNA cargo selection, content, release, and uptake. Cell Mol Neurobiol.

[3] Raposo G, Stahl PD (2019). Extracellular vesicles: a new communication paradigm?. Nat Rev Mol Cell Biol.

[4] García-Silva S, Gallardo M, Peinado H (2020). DNA-loaded extracellular vesicles in liquid biopsy: tiny players with big potential?. Front Cell Dev Biol.

[5] Zayakin P, Sadovska L, Eglītis K (2023). Extracellular vesicles - a source of RNA biomarkers for the detection of breast cancer in liquid biopsies. Cancers.

[6] Wang W, Qiao S, Kong X, Zhang G, Cai Z (2025). The role of exosomes in immunopathology and potential therapeutic implications. Cell Mol Immunol.

[7] Minciacchi VR, Freeman MR, Di Vizio D (2015). Extracellular vesicles in cancer: exosomes, microvesicles and the emerging role of large oncosomes. Semin Cell Dev Biol.

[8] Rondelli V, Helmy S, Passignani G, Parisse P, Di Silvestre D (2022). Integrated strategies for a holistic view of extracellular vesicles. ACS Omega.

[9] Sohal IS, Kasinski AL (2023). Emerging diversity in extracellular vesicles and their roles in cancer. Front Oncol.

[10] Doyle LM, Wang MZ (2019). Overview of extracellular vesicles, their origin, composition, purpose, and methods for exosome isolation and analysis. Cells.

[11] Théry C, Witwer KW, Aikawa E (2018). Minimal information for studies of extracellular vesicles 2018 (MISEV2018): a position statement of the International Society for Extracellular Vesicles and update of the MISEV2014 guidelines. J Extracell Vesicles.

[12] Jeppesen DK, Zhang Q, Franklin JL, Coffey RJ (2023). Extracellular vesicles and nanoparticles: emerging complexities. Trends Cell Biol.

[13] Raposo G, Stoorvogel W (2013). Extracellular vesicles: exosomes, microvesicles, and friends. J Cell Biol.

[14] Crescitelli R, Lässer C, Lötvall J (2021). Isolation and characterization of extracellular vesicle subpopulations from tissues. Nat Protoc.

[15] Elsharkasy OM, Nordin JZ, Hagey DW (2020). Extracellular vesicles as drug delivery systems: why and how?. Adv Drug Deliv Rev.

[16] Malhotra S, Miras MCM, Pappolla A, Montalban X, Comabella M (2023). Liquid biopsy in neurological diseases. Cells.

[17] Wiklander OPB, Brennan MÁ, Lötvall J, Breakefield XO, El Andaloussi S (2019). Advances in therapeutic applications of extracellular vesicles. Sci Transl Med.

[18] Hallal S, Ebrahimkhani S, Shivalingam B, Graeber MB, Kaufman KL, Buckland ME (2019). The emerging clinical potential of circulating extracellular vesicles for non-invasive glioma diagnosis and disease monitoring. Brain Tumor Pathol.

[19] Wang R, Wang X, Zhang Y (2022). Emerging prospects of extracellular vesicles for brain disease theranostics. J Control Release.

[20] Korte B, Mathios D (2024). Innovation in non-invasive diagnosis and disease monitoring for meningiomas. Int J Mol Sci.

[21] Cui L, Zheng J, Lu Y (2024). New frontiers in salivary extracellular vesicles: transforming diagnostics, monitoring, and therapeutics in oral and systemic diseases. J Nanobiotechnology.

[22] Fu Y, Zhang Y, Khoo BL (2021). Liquid biopsy technologies for hematological diseases. Med Res Rev.

[23] Sharma N, Angori S, Sandberg A (2024). Defining the soluble and extracellular vesicle protein compartments of plasma using in-depth mass spectrometry-based proteomics. J Proteome Res.

[24] Nieuwland R, Siljander PR (2024). A beginner’s guide to study extracellular vesicles in human blood plasma and serum. J Extracell Vesicles.

[25] Karimi N, Cvjetkovic A, Jang SC (2018). Detailed analysis of the plasma extracellular vesicle proteome after separation from lipoproteins. Cell Mol Life Sci.

[26] Tan Y, Kao WC, Boppart M, Sweedler JV (2025). Plasma-derived extracellular vesicle proteomics. J Proteome Res.

[27] Tóth EÁ, Turiák L, Visnovitz T (2021). Formation of a protein corona on the surface of extracellular vesicles in blood plasma. J Extracell Vesicles.

[28] de Menezes-Neto A, Sáez MJ, Lozano-Ramos I (2015). Size-exclusion chromatography as a stand-alone methodology identifies novel markers in mass spectrometry analyses of plasma-derived vesicles from healthy individuals. J Extracell Vesicles.

[29] Liangsupree T, Multia E, Riekkola ML (2021). Modern isolation and separation techniques for extracellular vesicles. J Chromatogr A.

[30] Damianidou E, Mouratidou L, Kyrousi C (2022). Research models of neurodevelopmental disorders: the right model in the right place. Front Neurosci.

[31] Vandamme T (2014). Use of rodents as models of human diseases. J Pharm Bioallied Sci.

[32] Rikkert LG, Coumans FAW, Hau CM, Terstappen LWMM, Nieuwland R (2021). Platelet removal by single-step centrifugation. Platelets.

[33] Ahmadian S, Jafari N, Tamadon A, Ghaffarzadeh A, Rahbarghazi R, Mahdipour M (2024). Different storage and freezing protocols for extracellular vesicles: a systematic review. Stem Cell Res Ther.

[34] Fratini F, Tamarozzi F, Macchia G (2020). Proteomic analysis of plasma exosomes from Cystic Echinococcosis patients provides *in vivo* support for distinct immune response profiles in active vs inactive infection and suggests potential biomarkers. PLoS Negl Trop Dis.

[35] (2016). Groot Kormelink T, Arkesteijn GJ, Nauwelaers FA, van den Engh G, Nolte-’t Hoen EN, Wauben MH. Prerequisites for the analysis and sorting of extracellular vesicle subpopulations by high-resolution flow cytometry. Cytometry A.

[36] Federici C, Shahaj E, Cecchetti S (2020). Natural-killer-derived extracellular vesicles: immune sensors and interactors. Front Immunol.

[37] Tyanova S, Temu T, Cox J (2016). The MaxQuant computational platform for mass spectrometry-based shotgun proteomics. Nat Protoc.

[38] Tusher VG, Tibshirani R, Chu G (2001). Significance analysis of microarrays applied to the ionizing radiation response. Proc Natl Acad Sci U S A.

[39] (2017). Van Deun J, Mestdagh P, Agostinis P, et al; EV-TRACK Consortium. EV-TRACK: transparent reporting and centralizing knowledge in extracellular vesicle research. Nat Methods.

[40] Webber J, Clayton A (2013). How pure are your vesicles?. J Extracell Vesicles.

[41] Cifola I, Fratini F, Cardinali B (2022). miRNome and proteome profiling of small extracellular vesicles secreted by human glioblastoma cell lines and primary cancer stem cells. Biomedicines.

[42] Robinson SD, de Boisanger J, Pearl FMG, Critchley G, Rosenfelder N, Giamas G (2024). A brain metastasis liquid biopsy: where are we now?. Neurooncol Adv.

[44] Diaz Lozano IM, Sork H, Stone VM (2022). Proteome profiling of whole plasma and plasma-derived extracellular vesicles facilitates the detection of tissue biomarkers in the non-obese diabetic mouse. Front Endocrinol.

[45] Baranyai T, Herczeg K, Onódi Z (2015). Isolation of exosomes from blood plasma: qualitative and quantitative comparison of ultracentrifugation and size exclusion chromatography methods. PLoS One.

[46] Michell DL, Allen RM, Landstreet SR (2016). Isolation of high-density lipoproteins for non-coding small rna quantification. J Vis Exp.

[47] Vallejo MC, Sarkar S, Elliott EC (2023). A proteomic meta-analysis refinement of plasma extracellular vesicles. Sci Data.

[48] Sidhom K, Obi PO, Saleem A (2020). A review of exosomal isolation methods: is size exclusion chromatography the best option?. Int J Mol Sci.

[49] Takov K, Yellon DM, Davidson SM (2019). Comparison of small extracellular vesicles isolated from plasma by ultracentrifugation or size-exclusion chromatography: yield, purity and functional potential. J Extracell Vesicles.

[50] Mol EA, Goumans MJ, Doevendans PA, Sluijter JPG, Vader P (2017). Higher functionality of extracellular vesicles isolated using size-exclusion chromatography compared to ultracentrifugation. Nanomedicine.

[51] Robinson SD, Samuels M, Jones W (2024). Confirming size-exclusion chromatography as a clinically relevant extracellular vesicles separation method from 1mL plasma through a comprehensive comparison of methods. BMC Methods.

[52] Zhang X, Takeuchi T, Takeda A, Mochizuki H, Nagai Y (2022). Comparison of serum and plasma as a source of blood extracellular vesicles: increased levels of platelet-derived particles in serum extracellular vesicle fractions alter content profiles from plasma extracellular vesicle fractions. PLoS One.

[53] Małys MS, Köller MC, Papp K (2023). Small extracellular vesicles are released ex vivo from platelets into serum and from residual blood cells into stored plasma. J Extracell Biol.

[54] Davidson SM, Takov K, Yellon DM (2017). Exosomes and cardiovascular protection. Cardiovasc Drugs Ther.

[55] Brennan K, Martin K, FitzGerald SP (2020). A comparison of methods for the isolation and separation of extracellular vesicles from protein and lipid particles in human serum. Sci Rep.

[56] Hirschberg Y, Boonen K, Schildermans K (2022). Characterising extracellular vesicles from individual low volume cerebrospinal fluid samples, isolated by SmartSEC. J Extracell Biol.

[57] Liam-Or R, Faruqu FN, Walters A (2024). Cellular uptake and in vivo distribution of mesenchymal-stem-cell-derived extracellular vesicles are protein corona dependent. Nat Nanotechnol.

[58] Heidarzadeh M, Zarebkohan A, Rahbarghazi R, Sokullu E (2023). Protein corona and exosomes: new challenges and prospects. Cell Commun Signal.

[59] Arredondo-Damián JG, Martínez-Soto JM, Molina-Pelayo FA (2024). Systematic review and bioinformatics analysis of plasma and serum extracellular vesicles proteome in type 2 diabetes. Heliyon.

