# Supplementary material for: Improvement of small extracellular vesicle isolation from mouse model blood
Source: Extracell Vesicles Circ Nucl Acids. 2025 Dec 24;6(4):1034–53. doi: 10.20517/evcna.2025.90 (PMC12812443; doi:10.20517/evcna.2025.90)
Supplement: Supplementary file 1 [file evcna-6-4-1034-SupplementaryMaterials.zip › evcna6090-SupplementaryMaterials/evcna6090-SupplementaryMaterials.pdf]

Supplementary Materials

Improvement of small extracellular vesicle isolation from mouse model blood

Gloria Venturini[1,2], Antonella Ferrante[1], Nazzareno Di Carlo[1], Lucia Bertuccini[3], Francesca Iosi[3], Maria Condello[1], Alberto Martire[1], Federica Fratini[4], Zaira Boussadia[1]

[1]National Centre for drug research and evaluation, Italian National Institute of Health, Rome 00161, Italy.
[2]Department of Life and Environmental Sciences, Polytechnic University of Marche, Ancona 60131, Italy.
[3]Core Facilities, Microscopy Area, Italian National Institute of Health, Rome 00161, Italy.
[4]Department of Neurosciences, Italian National Institute of Health, Rome 00161, Italy.

Correspondence to: Dr. Federica Fratini, Department of Neurosciences, Italian National Institute of Health, Rome 00161, Italy. E-mail: federica.fratini@iss.it

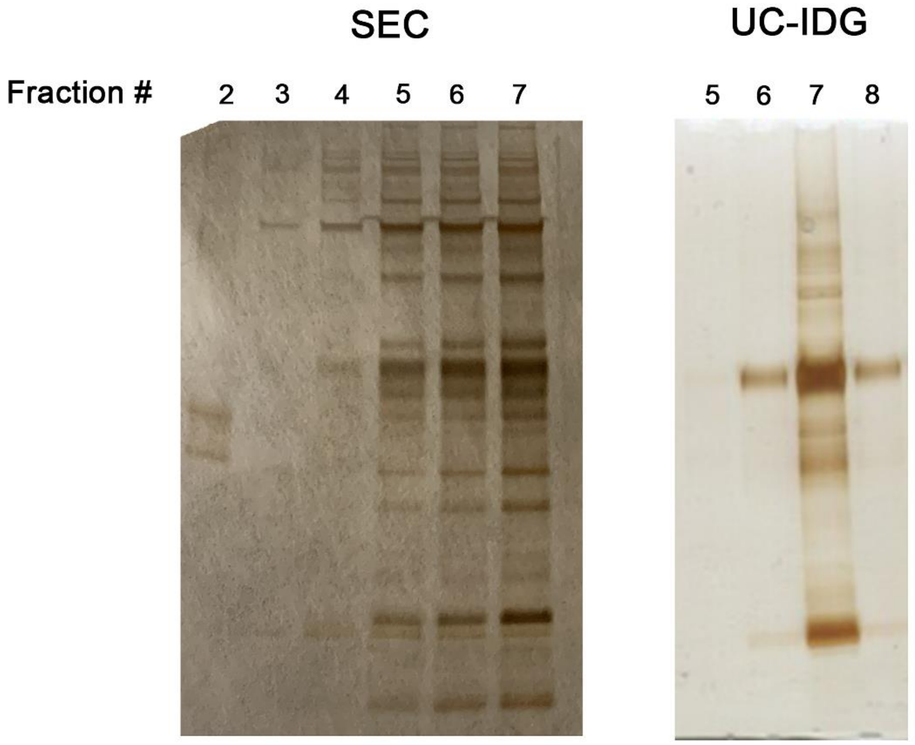

Supplementary Figure 1. Silver staining of SEC and UC-IDG fractions for protein profile visualization. 10 µL of each fraction were resolved by SDS-Polyacrylamide gel electrophoresis. Silver staining was performed following the manufacturer's instructions (Thermo Scientific). The gel shows the protein profile of SEC and UC-IDG fractions.

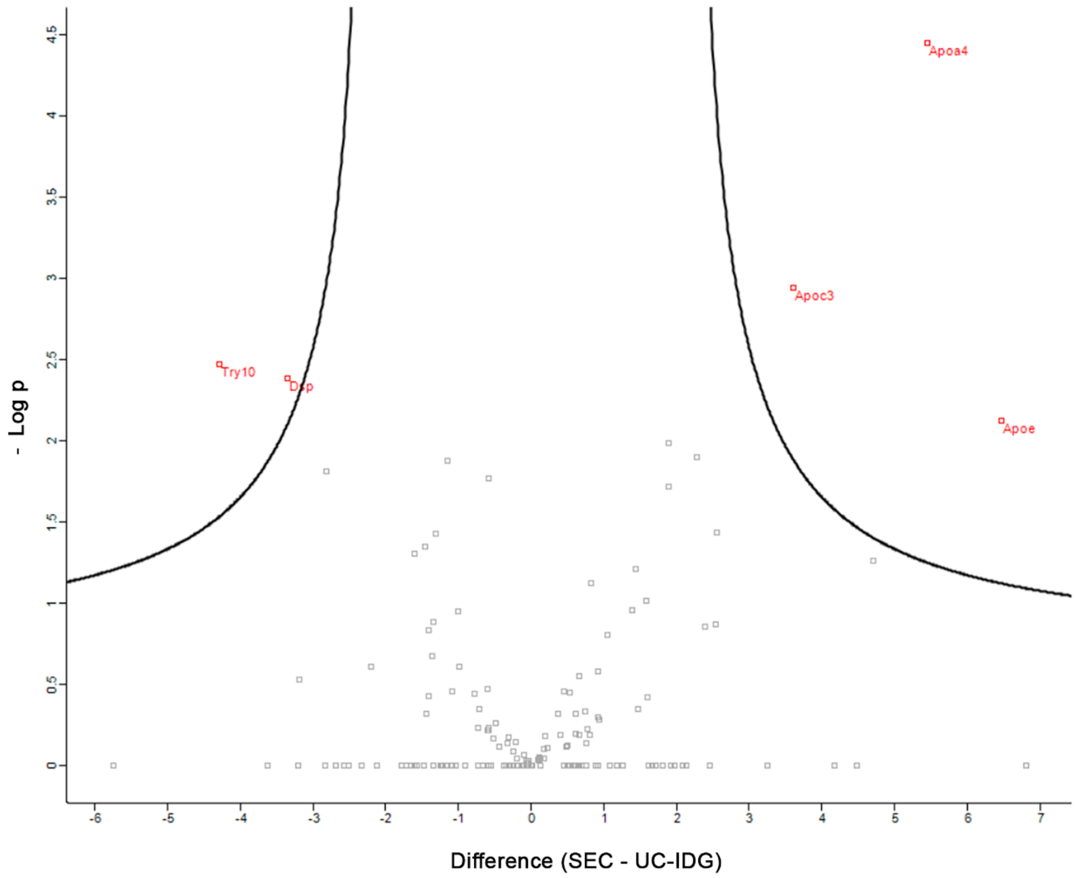

Supplementary Figure 2. Volcano plot showing differentially enriched proteins between the two isolation methods. Two-sample Student's *t*-test was performed using the Perseus platform (S0 = 1.5 and FRD = 0.01). The statistical comparison shows no differences in terms of quantitative abundance of proteins identified in the two samples, if not for few apoliproteins (A4, C3, E) known to be part of the vesicle-corona in SEC sample and blood contaminants (Trypsin and Desmoplakin) in UC-IDG sample.

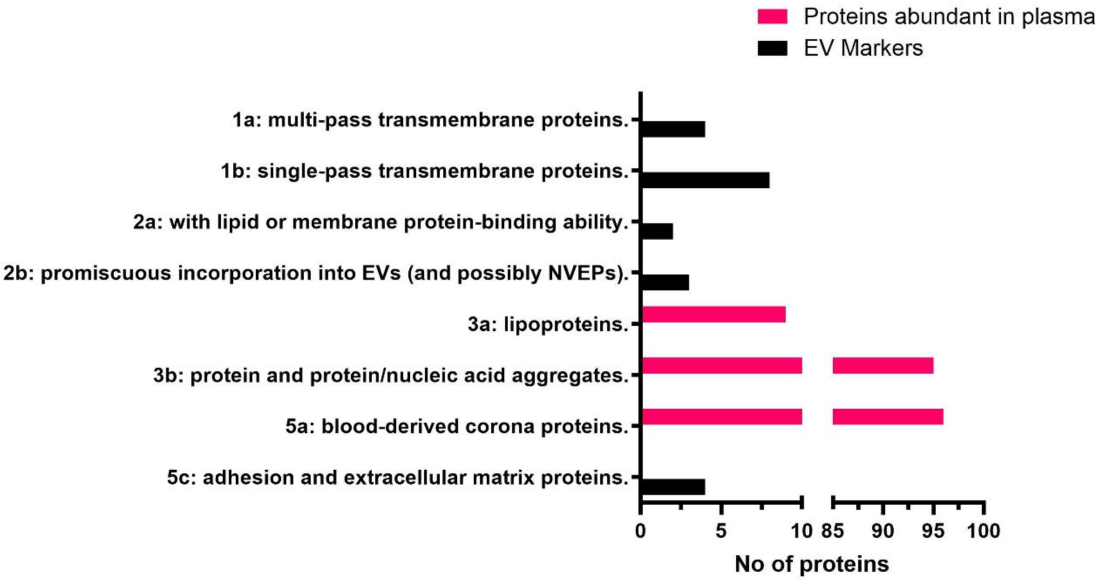

Supplementary Figure 3. Validation of initial protein classification through MISEV categorization. Bar chart showing the distribution of identified proteins across MISEV2023 categories. Proteins initially annotated as "EV markers" and "proteins abundant in plasma" were assigned to MISEV2023 categories to gain insight into the initial provided protein categorization in Figure 4B.
